# Left ventricular diastolic function and cardiotoxic chemotherapy

**DOI:** 10.1186/s43044-024-00476-4

**Published:** 2024-04-12

**Authors:** Haider Rashid, Aamir Rashid, Asif Mattoo, Faisal R. Guru, Syed Mehvish, Shahood Ajaz Kakroo, Ajaz Ahmad Lone, Khursheed Aslam, Imran Hafeez, Hilal Rather

**Affiliations:** 1 Department of Cardiology, SKIMS, Soura, Srinagar, J & K India; 2 Department of Medical Oncology (Paediatric Unit), SKIMS, Soura, Srinagar, J & K India; 3 Department of Psychiatry, GMC Srinagar, Srinagar, J & K India

**Keywords:** Diastolic dysfunction, Cardiotoxic chemotherapy, Left ventricular systolic dysfunction, Global longitudinal strain

## Abstract

**Background:**

Left ventricular ejection fraction falls when the myocardium has already lost a significant portion of its functional capacity. There are conflicting data on whether diastolic dysfunction precedes systolic dysfunction after cardiotoxic chemotherapy. We aimed to study systolic and diastolic dysfunction after cardiotoxic chemotherapy and whether diastolic dysfunction can predict subsequent risk of systolic dysfunction. It was an observational prospective cohort study, and patients receiving cardiotoxic chemotherapy were included. Baseline, demographic, and clinical details were recorded. Echocardiographic measurements of left ventricular systolic function, global longitudinal strain, and diastolic function were noted at baseline, three months, and 6 months.

**Results:**

We included eighty patients. The mean age of the patients was 54.92 ± 7.6 years, predominantly females (80%). The mean left ventricular ejection fraction fell from 64.92 ± 1.96 to 60.97 ± 4.94 at 6 months. Low ejection fraction was seen in 8 (10%) patients at 6 months. The mean global longitudinal strain (GLS) at baseline was − 18.81 ± 0.797 and fell to − 17.65 ± 2.057 at 6 months, with 12 (15%) patients having low GLS (< − 18). Grade 1 diastolic dysfunction was seen in 22 (27.5%) patients, and grade 2 diastolic dysfunction was seen in 3 (3.8%) patients at 6 months. There was a significant decrease in *E*/*A* ratio (inflow early diastolic velocity/Inflow late diastolic velocity), mitral tissue Doppler velocity, and an increase in isovolumic relaxation time, mitral valve deceleration time, and *E*/*e*′ (inflow early diastolic velocity/tissue Doppler mitral annular velocity), at three months and 6 months. Ejection fraction at 6 months was significantly and negatively correlated with diastolic dysfunction at three months (*r* = − 0.595, *p* = 0.02).

**Conclusions:**

Cardiotoxic chemotherapy is associated with early diastolic dysfunction. Early diastolic dysfunction predicts subsequent left ventricular systolic dysfunction.

## Background

Progress in early detection and treatment has improved the outlook for cancer patients [1]. Yet, chemotherapy's cardiotoxic effects significantly contribute to enduring health challenges and even fatalities [2, 3]. Long-term cancer survivors who have received cardio toxic chemotherapy are at increased risk of cardiac events [4]. Anthracyclines induces apoptosis of cardiomyocytes, oxidative stress and, mitochondrial dysfunction leading to left ventricular dysfunction [6, 7]. Cardio toxicity is seen in nine percent of patients receiving anthracyclines [8].

The assessment of cardiac function during chemotherapy primarily relies on measuring the left ventricular ejection fraction (LVEF) by echocardiography. Although commonly accepted, it can only diagnose left ventricular dysfunction only when the myocardium has already lost a significant portion of its functional capacity. As a result, it serves as a relatively late indicator of left ventricular dysfunction [9]. It is paramount to identify dependable, non-invasive techniques for the early detection of myocardial dysfunction before reaching the critical stage of left ventricular dysfunction.

Detecting alterations in cardiac parameters before a reduction in LVEF occurs is pivotal for identifying patients who could benefit from early cardioprotective interventions. This, in turn, significantly decreases the probability of interrupting effective systemic treatment, potentially jeopardizing patients' long-term well-being. Though different methods are utilized to detect early cardiotoxicity, there remains an unmet need regarding the most ideal way to detect early cardiotoxicity.

The pathogenesis of heart failure involves significant diastolic alterations [10, 11]. Diastolic dysfunction and its severity on echocardiography are associated with risk of overt heart failure on follow-up [12].

Prior research has shown that anthracyclines cause diastolic alterations. However, there are conflicting data on whether diastolic dysfunction precedes systolic dysfunction. While some studies [13, 14] have shown that diastolic dysfunction does not predate systolic dysfunction, other studies [15–21] have shown that abnormal diastology precedes systolic dysfunction. We designed this prospective study to understand how common diastolic dysfunction is after cardiotoxic chemotherapy and whether diastolic dysfunction can predict subsequent risk of systolic dysfunction.

## Methods

### Study design

Our longitudinal prospective cohort study was conducted over 24 months at our tertiary care hospital in the Department of Cardiology and Medical Oncology. The study was approved by the Institute Ethics Committee (IEC) with reference number of SIMS 131/IEC-SKIMS/2019-356. All patients provided written informed consent.

### Study subjects

The inclusion criteria were: patients receiving cardiotoxic chemotherapy, expected survival of more than 6 months, age ≥ 18 years, normal left ventricular (LV) ejection fraction (≥ 52% for men and ≥ 54% for women) [22], with normal ECG, normal LV diastolic function, and those who gave written informed consent. The exclusion criteria were those with left ventricular (LV) systolic dysfunction, LV diastolic dysfunction of ≥ Grade 1 at baseline, suboptimal echocardiographic assessment, who have received chemotherapy in the past, current pregnancy, and breastfeeding. From cardiological point of view, we excluded breastfeeding women due to concerns about lactation-related physiological changes including fluctuations in fluid balance, electrolyte levels, and hormonal fluctuations, which could affect cardiac function and complicate the interpretation of echocardiographic results.

### Clinical characteristics

We recruited cases from outpatient and inpatient medical oncology departments. Clinical details that were noted include the type of malignancy, chemotherapy used, clinical stage, hormone receptor status, any comorbidities (hypertension, diabetes, dyslipidemia, tobacco use, obesity), radiation treatment, and surgical details. Hypertension was determined by the 2018 American Heart Association/American College of Cardiology (AHA/ACC) guidelines [23]. Diabetes was characterized by a fasting blood glucose level greater than 126 mg/dl or an HbA1C level of 6.5 or higher or if the patient was already being treated for diabetes mellitus. Dyslipidemia was defined by the presence of any one of the following: LDL > 130 mg/dl, Total cholesterol > 200 mg/dl, and HDL < 40 mg/dl in men and < 50 mg/dl in women.

### Echocardiographic assessment

A baseline and follow-up echocardiograms were recorded at three months and 6 months. Transthoracic echocardiograms were performed in the cardiology department on GE Vivid E 95 machines (GE Healthcare, Milwaukee, Wisconsin). Echocardiography included assessment of the following parameters: Left ventricular ejection fraction (LVEF) was measured by modified Simpson’s method. Global longitudinal strain (GLS) was measured in the standard way [22]. The diastolic function parameters included mitral E velocity (mitral inflow early diastolic velocity), mitral A velocity (mitral inflow late diastolic velocity), E/A ratio, *e*′ (tissue Doppler mitral annular velocity), average E/e,’ mitral valve deceleration time (DT), isovolumic relaxation time (IVRT), peak tricuspid regurgitation (TR) systolic jet velocity. The left atrial (LA) volume index was determined by employing the both the 4-chamber and 2-chamber apical views just before the mitral valve (MV) opened. This calculation utilized the area-length method and was adjusted by dividing it by the individual's body surface area (BSA). Diastolic dysfunction was graded as per ASE guidelines [22]. Cardiac toxicity was diagnosed as a fall of more than ten percent in LVEF without any symptoms or more than a five percent decrease in LVEF along with the presence of heart failure symptoms [24]. Two experienced echocardiographers evaluated the echo cardio graphic parameters. The use of percentages to report the mean value was reported to give the coefficient of variation, which was calculated for intra-observer and inter-observer variability. The intra-observer coefficient of variation (CV) for LVEF was 2.4%. The inter-observer CV for LVEF was 3.4%. The intra-observer CV for GLS was 2.8%. The inter-observer CV for LVEF was 3.2%. The CV (both for intra- and inter-observer) for other diastolic echocardiographic parameters was less than 5%.

### Statistical analysis

Sampling was done by purposive sampling technique. The calculation of required sample size was done using Open Epi statistical software version 3 using the following assumptions of a single population proportion formula for the descriptive studies; Prevalence of chemotherapy associated left ventricular dysfunction 6%, with confidence limit of 95% and precision, which was set at 0.05. Thus, the sample came out to be 77. We recruited a total of 80 patients. The data were analyzed by Statistical Package for Social Sciences (SPSS Version 20.0, SPSS Inc., Chicago, Illinois, USA). Continuous variables were expressed as mean ±standard deviation and categorical variables as frequencies and percentages. Pearson’s Chi-square was used for inferential statistics, two-sided *p* values were reported, and a *P* value of less than 0.05 was considered statistically significant.

## Results

### Baseline characteristics

Initially, ninety patients were enrolled. However, five patients were excluded at baseline due to suboptimal echocardiographic assessment of diastolic dysfunction, and another five patients were lost to follow-up. So, a total of eighty patients were included in the final analysis. The mean age of the patients was 54.92 ± 7.6 years, predominantly females (80%). The baseline clinical, demographic, and echocardiographic variables are shown in Table 1. All baseline echocardiographic variables were within normal range. The different type of malignancies included 43 (53.75%) Her 2-positive breast cancer cases, 17 (21.25%), Her 2-negative breast cancer cases, and 20 (25%) Hodgkins lymphoma cases. The cumulative dose of anthracycline was 323.40 ± 101.34 mg/m^2^ (127.51–430.53 mg/m^2^).

**Table 1:** Baseline clinical, demographic, and echocardiographic variables

Age (years)	54.92 ± 7.6
Female (%)	64 (80%)
BMI (kg/m^2^)	24.7 ± 3.5
Hypertension	46 (57.5%)
Diabetes	38 (47.5%)
Smoking	13 (16.25%)
Radiation therapy	42 (52.5%)
Stage	
1	14 (17.5%)
2	48 (60%)
3	17 (21.25%)
4	1 (1.25%)
Echocardiographic parameters	Mean ± SD
Ejection fraction	64.92 ± 1.96
*E*/*A*	1.36 ± 0.138
DT (ms)	179.62 ± 8.92
IVRT (ms)	86.92 ± 4.53
*e*′ septal	10.6 ± 0.83
E ‘lateral	14.01 ± 0.806
*E*/*e*′ septal	9.21 ± 0.731
Peak TR velocity m/s	2.12 ± 0.287
GLS	− 18.81 ± 0.797

*E* mitral inflow early diastolic velocity; *A* mitral inflow late diastolic velocity; *DT* deceleration time; *IVRT* isovolumic relaxation time; *e*′ mitral annular early tissue Doppler velocity; *TR* tricuspid regurgitation; *GLS* global longitudinal strain; *MS* mill seconds. Stage 1 localized cancer; Stage 2 early locally advanced; Stage 3 late locally advanced

Stage 4 metastasis

### Changes in diastolic function

**Fig. 1:**
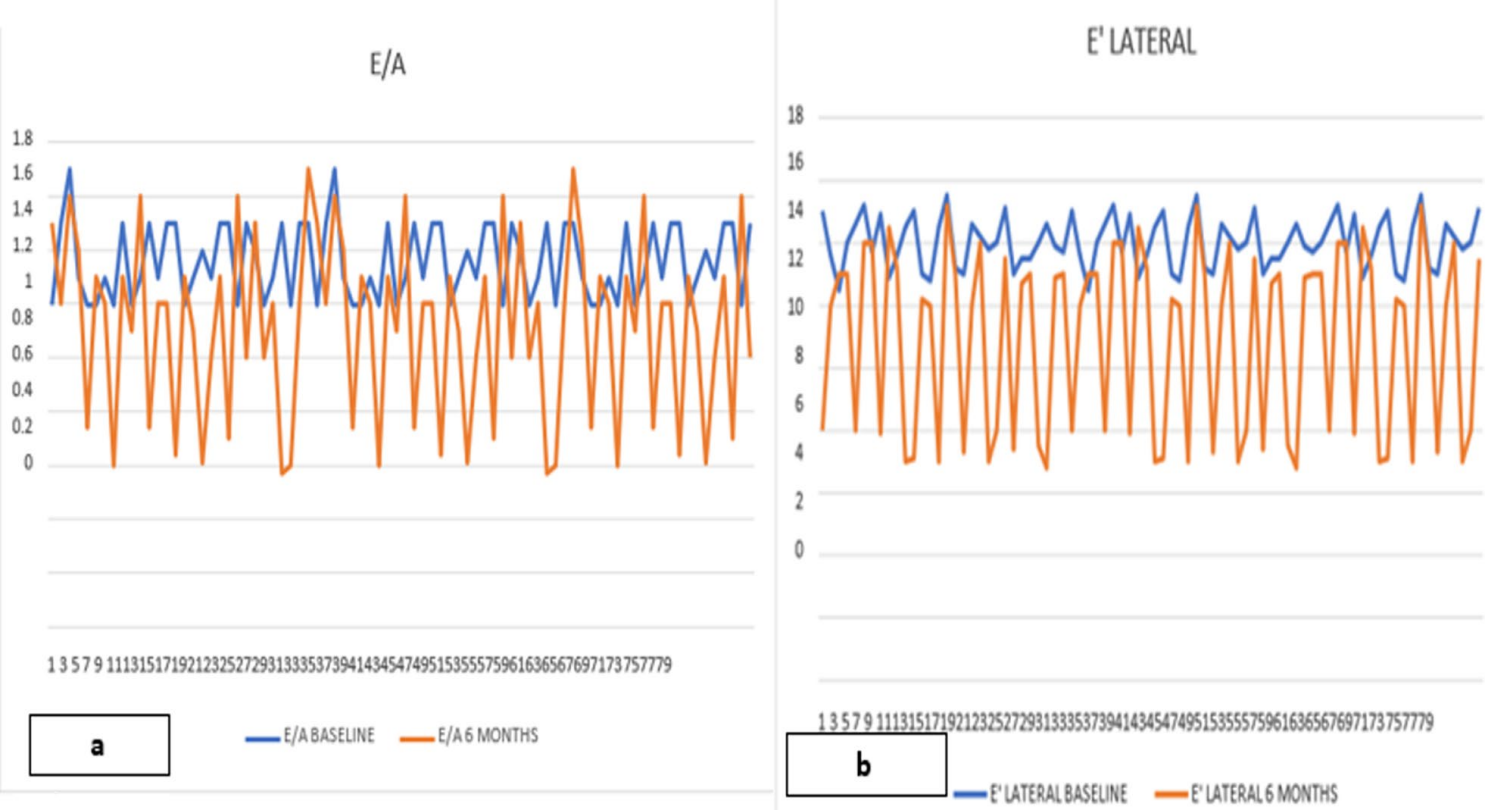
**a** Comparison of E/A at baseline and 6 months. **b** Comparison lateral tissue Doppler mitral annular velocity baseline and 6 months. E = inflow early diastolic velocity; A = inflow late diastolic velocity; e*′* tissue Doppler mitral annular velocity

**Fig. 2:**
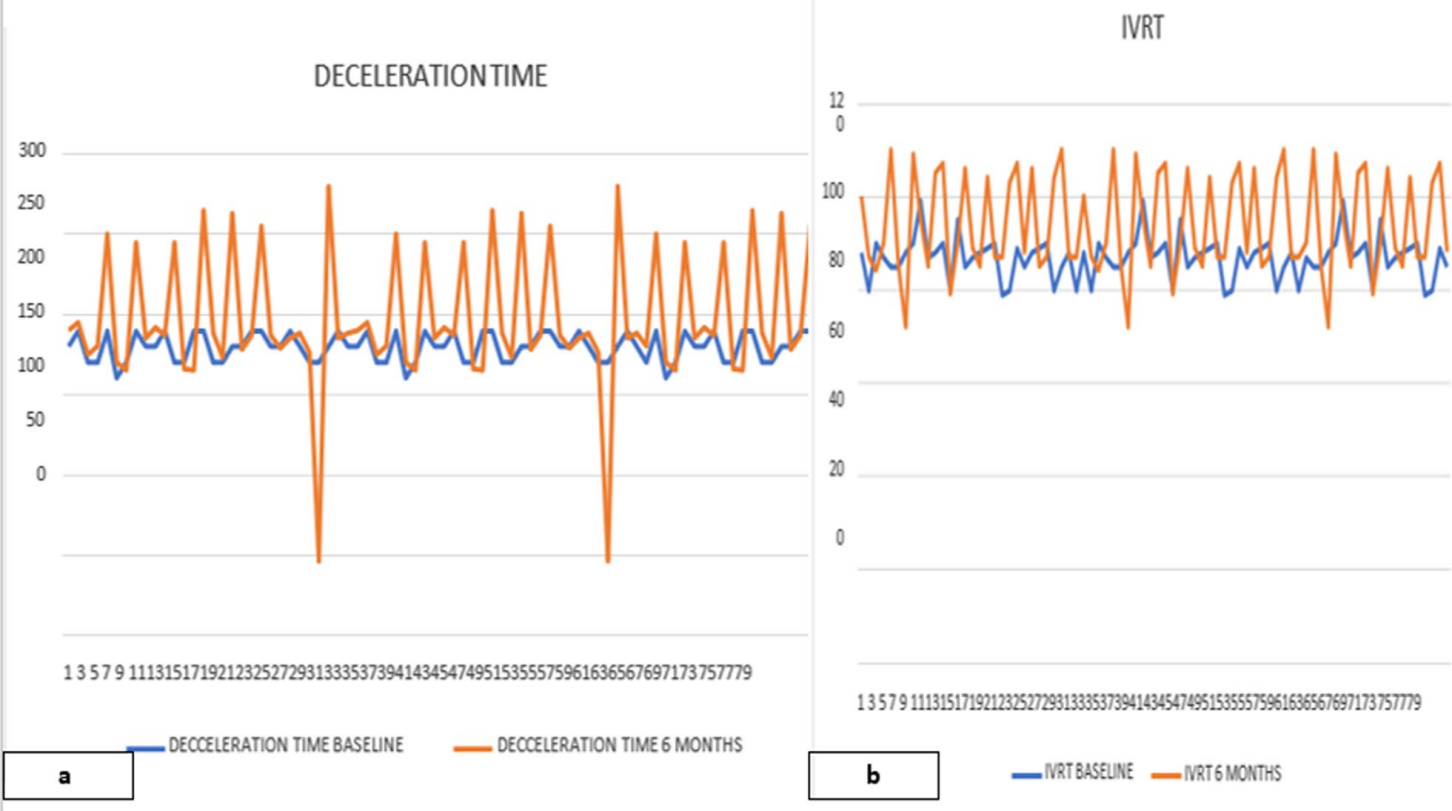
**a** Comparison of deceleration time at baseline and 6 months. **b:** comparison Isovolumic relaxation time at baseline and 6 months. IVRT isovolumic relaxation time

At 6 months of follow-up, grade 1 diastolic dysfunction was seen in 22 (27.5%) patients, and grade 2 diastolic dysfunction was seen in 3 (3.8%) patients. The remaining 55 (68.8%) of patients had no diastolic dysfunction. The mean E/A ratio decreased significantly over 6 months (Fig. 1a). There were also a modest significant reduction in lateral and septal *e*′ (Fig. 1b) and an increase in the *E*/*e*′ ratio. LA volume index also increased significantly at 6 months (Table 2). The DT and IVRT (Fig. 2a, b) also increased significantly over time (Table 2). The logistic regression analysis showed radiation therapy, age ≥ 50 years and BMI ≥ 27 kg/m^2^ to be significantly associated with occurrence of diastolic dysfunction. However, there was no association with diabetes mellites, tobacco use or hypertension with diastolic dysfunction (Table 3).

**Table 2:** Baseline and follow-up echocardiographic parameters

Parameter	Baseline	At 3 months	At 6 months	*P* value (*)	*P* value (**)
	Mean ± SD	Mean ± SD	Mean ± SD		
Ejection fraction	64.92 ± 1.96	64.44 ± 1.56	60.97 ± 4.94	0.08	*P* = 0.0001
E/A	1.36 ± 0.138	1.20 ± 0.343	1.11 ± 0.333	0.0002	*P* = 0.0001
DT (ms)	179.62 ± 8.92	185.23 ± 11.21	196.14 ± 41.94	0.0006	*P* = 0.0001
IVRT (ms)	86.92 ± 4.53	91.82 ± 6.54	94.04 ± 10.70	0.0001	*P* = 0.0001
*e*′ septal	10.6 ± 0.83	9.58 ± 1.06	8.58 ± 2.07	0.0001	*P* = 0.0001
e ‘lateral	14.01 ± 0.806	12.04 ± 1.96	10.90 ± 2.96	0.0001	*P* = 0.0001
*E*/*e*′ septal	8.66 ± 1.085	9.01 ± 0.645	9.21 ± 0.731	0.01	*P* = 0.0002
Peak TR velocity m/s	2.12 ± 0.287	2.20 ± 0.590	2.22 ± 0.390	0.27	*P* = 0.06
LAVI (ml/BSA)	21.59 ± 2.29	21.89 ± 2.25	24.56 ± 2.15	0.34	0.001
GLS	− 18.81 ± 0.797	− 18.26 ± 1.7	− 17.65 ± 2.057	0.009	*P* = 0.0001

*E* mitral inflow early diastolic velocity; *A* mitral inflow late diastolic velocity; *DT* deceleration time; *IVRT* isovolumic relaxation time; *e*′ mitral annular early tissue Doppler velocity; *TR* tricuspid regurgitation; *GLS* global longitudinal strain; *LAVI* (ml/BSA) left atrial volume index/body surface area

P* = *P* value between baseline and three months

P** = *P* value between baseline and at 6 months

**Table 3:** Factors affecting the occurrence of diastolic dysfunction: logistic regression analysis results

Factor	Odds ratio	95% CI	*p* value
Smoking	1.11	(1.05, 1.48)	0.090
Hypertension	1.14	(1.35, 3.25)	0.310
Diabetes	1.15	(0.90, 1.42)	0.320
Radiation	1.70	(1.15, 2.78)	0.012
Age ≥ 50 years	1.50	(1.25, 1.85)	0.001
BMI ≥ 27 kg/m^2^	1.60	(1.15, 2.55)	0.001

### Changes in left ventricular systolic function and GLS.

**Fig. 3:**
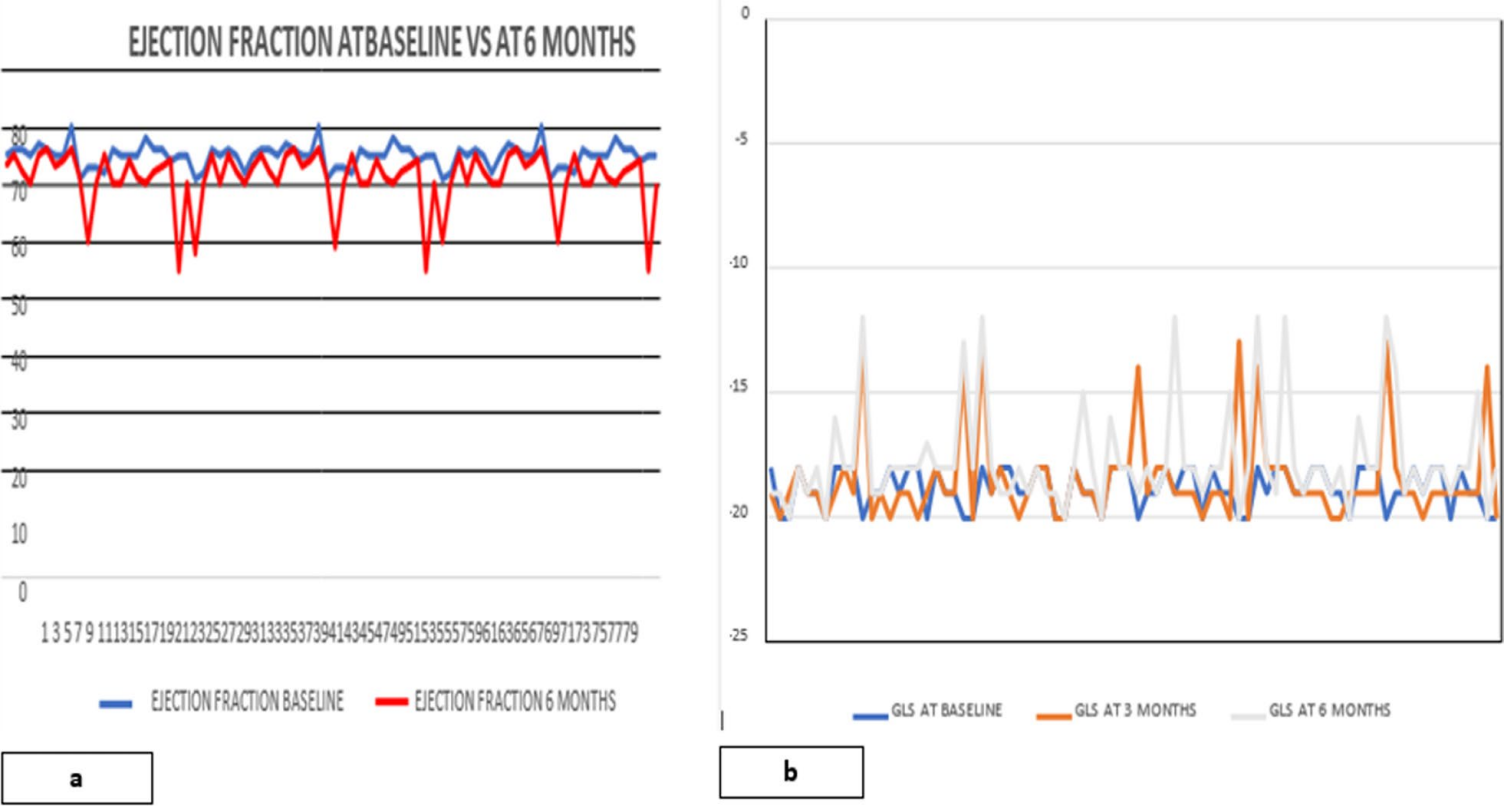
**a** Comparison of ejection fraction at baseline and 6 months. **b** Comparison of Global Longitudinal strain at baseline and 6 months. GLS global longitudinal strain

Low ejection fraction (systolic dysfunction) was seen in 8 (10%) patients at 6 months. The mean LVEF fell from 64.92 ± 1.96 to 60.97 ± 4.94 at 6 months (Fig. 3a). The mean global longitudinal strain (GLS) at baseline was − 18.81 ± 0.797. At three months, mean GLS reduced to − 18.46 ± 1.793, with 9 (11.25%) patients having low GLS (< − 18). At 6 months, a significant decrease in mean GLS was noted, i.e., 17.65 ± 2.057, with 12 (15%) patients having low GLS (< − 18) (Fig. 3b).

### Association between diastolic dysfunction and systolic dysfunction

We analyzed whether diastolic dysfunction at three months had any correlation with the development of systolic dysfunction at 6 months. Pearson’s product moment of correlation analyses was applied. It was found that ejection fraction at 6 months was significantly and negatively correlated with diastolic dysfunction at three months (*r* = − 0.595, *p* = 0.02). LVEF at 6 months was inversely related to the diastolic dysfunction grade at three months. We also analyzed the association between LVEF and diastolic dysfunction at 6 months and found a significant correlation between the two at 6 months. (*r* = − 0.656, *p* = 0.01). The mean LVEF was significantly lower in those with grade ≥ 1 diastolic dysfunction than those without diastolic dysfunction (58.20 ± 6.819 vs. 62.60 ± 2.096) (*p* = 0.01). The mean difference of LVEF in patients having diastolic dysfunction Vs no diastolic dysfunction was -4.40 at 6 months.

### Relation between GLS and ejection fraction

There was no significant correlation between the GLS at and LVEF at three-month follow-up. (*r* = 0.195, *p* = 0.083). However, it was found that GLS at three months and LVEF at 6 months had positive correlation (*r* = 0.680, *p* < 0.01). Patients showing a fall in ejection fraction at 6 months had a prior decrease of GLS at three months (Table 4).

**Table 4:** Relationship between global longitudinal strain at 3 months and LVEF at 6 months

GLS at 3 months	Ejection fraction at 6 months (mean ± SD)	Number
− 20	62.29 ± 2.312	17
− 19	62.56 ± 2.036	39
− 18	62.31 ± 2.469	16
− 14	47.25 ± 2.630	4
− 13	48.25 ± 2.363	4
Total	60.97 ± 4.943	80

*GLS* global longitudinal strain; *LVEF* left ventricular ejection fraction

## Discussion

The present study evaluated the left ventricular diastolic function parameters and their relationship with systolic dysfunction in cancer chemotherapy patients. Our salient findings include: (1) There is early worsening of diastolic dysfunction, and it persists over time. (2) The early diastolic dysfunction is associated with subsequent systolic dysfunction. (3) Ten percent of our patients developed significant fall in LVEF over 6 months.

Around one-third of our patients showed evidence of diastolic dysfunction at 6 months. The E/A ratio decreased over three months and worsened up to 6 months of follow-up. Both DT and IVRT increased as early as three months, with a sustained modest decrease at 6 months. The tissue Doppler velocities also decreased significantly. *E*/*e*′ showed a modest increase in follow-up. Our study showed an initial E/A ratio average of 1.36, decreasing to 1.11 after 6 months. This aligns with findings from Upshaw et al. [15], (baseline E/A ratio: 1.2), Boyd et al. [25], (baseline E/A ratio: 1.13), and Cochera et al. [26], (initial E/A ratio: 1.3), all demonstrating a decrease in E/A ratio over time. These consistent trends across studies suggest a pattern of declining E/A ratios in similar patient populations, highlighting a consistent pattern across different studies. Our study noted an increase in DT from 179 initially to 190 ms at the 6-month follow-up, consistent with observations reported by Serrano et al. [17], (186 to 201 ms). However, findings regarding IVRT varied among studies; Stoodley et al. [19], reported a significant increase in IVRT as early as one week, whereas Boyd et al. [25] found no significant change in IVRT during follow-up. The LA volume (ml/BSA) in our study also significantly increased from 21.59 ± 2.29 to 24.56 ± 2.15 at 6 months. However, it did not show any significant change at three months. Although it is a significant method for evaluating remodeling and indirectly assessing LA function, the indexed LA volume exhibits limited sensitivity during the initial phase [27]. Previous studies have shown that LA volume index may not change till 12 months of follow-up [28]. Similar findings were noted by Upshaw et al. [15]. A standard echocardiographic examination is incomplete without assessing diastology. Nevertheless, when monitoring for cardiotoxicity, the emphasis has traditionally been on assessing LV systolic function. Damage to the myocardial membrane and mitochondria has been observed after typical clinical doses of doxorubicin and could lead to impaired left ventricular relaxation and filling [29, 30]. The effects of doxorubicin on systolic and diastolic dysfunction vary between the early and late stages. Initially it can increase the systolic and diastolic function by increasing intramyocardial calcium levels or catecholamines [30–32]. However, on long term it decreases the systolic and diastolic dysfunction by direct myocardial injury. However, in children it has shown to acutely deteriorate systolic and diastolic function [20].

Our study population had a higher percentage of hypertensive patients (57.5%) and diabetic patients (47.5%) than other studies. This may be due to the high prevalence of diabetes and hypertension in our local population [33, 34]. Besides, sampling bias may also be contributory. Increasing age, BMI and radiotherapy were positively associated with diastolic dysfunction at 6 months. Age is an established risk factor for cardiotoxic chemotherapy [35, 36]. Increasing BMI has also been associated with worsening cardiac function following anthracycline treatment [37, 38]. Fumoleau et al. [37] found that a BMI of more than 27 kg/m^2^ significantly correlated with left ventricular dysfunction after epirubicin treatment, with an incidence of 1.8% versus 0.9% in patients with a BMI less than 27 kg/m^2^. The different mechanisms suggested include higher oxidative stress, high peripheral resistance, and chronic volume overload in obese patients. Radiation therapy is known to cause heart failure with preserved ejection fraction by myocardial fibrosis and left ventricular hypertrophy [39]. One recent study [40] found that radiation therapy is associated with diastolic dysfunction even in those without any systolic dysfunction as determined by GLS. There was no correlation with diabetes mellitus, tobacco use, or hypertension in our study subjects. We suggest the necessary measures to improve risk factors linked to diastolic dysfunction like regular physical activity and maintaining optimal weight. This can potentially lead to a delay in worsening of left ventricular dysfunction.

### Association with systolic dysfunction

Our study adds to growing body of knowledge about early impairment of diastolic dysfunction and its relation with subsequent systolic dysfunction. Diastolic dysfunction at three months significantly correlated with depression of ejection fraction at 6 months. LVEF at 6 months was inversely related to the diastolic dysfunction grade at three months. Diastolic impairment before a fall of LVEF is known to occur in coronary artery disease, diabetes, and hypertension [41, 42]. Though some studies [13, 14] have shown simultaneous impairment of left ventricular systolic and diastolic impairment, the most extensive study by Upshaw et al. [15] showed that worsening diastolic dysfunction is associated with increased systolic dysfunction on follow-up. The multicenter study by Calabrese et al. [21], reported LV diastolic dysfunction in 36% of patients as early as one week after starting chemotherapy. According to Stoddard et al. [29], an increase of more than 37% in IVRT demonstrated a sensitivity of 78% and a specificity of 88% for identifying the future development of doxorubicin-induced systolic dysfunction. The mechanism of early diastolic impairment is not yet clear. The probable hypothesis is that the left ventricular endocardium is more sensitive to microvascular abnormalities and interstitial fibrosis, resulting in early impairment of subendocardial function reflected by decreased left ventricular longitudinal strain and abnormal diastolic parameters [43, 44]. With the progression of the disease, the left ventricular ejection fraction also falls due to the involvement of mid-myocardial fibers [45].

### Global longitudinal strain and LVEF

Systolic dysfunction was seen in 8 (10%) patients at 6 months. The mean LVEF fell from 64.92 ± 1.96 to 60.97 ± 4.94 at 6 months. The mean global longitudinal strain (GLS) at baseline was − 18.81 ± 0.797 and fell to − 17.65 ± 2.057 at 6 months with 12 (15%) patients having low GLS (< − 18). Our findings are similar to those of Gripp et al. [46], who found that in breast cancer patients undergoing anthracycline and/or trastuzumab treatment, those developing cardiotoxicity showed a significant fall in mean left ventricular GLS compared to baseline at the third month. According to Negishi et al. [47], a reduction of over 11% in GLS indicated a long-term decrease in LVEF and cardiotoxicity in patients who received anthracyclines. Several hypotheses have been proposed to explain anthracycline-induced left ventricular dysfunction. The mechanisms implicated include oxidative stress, lipid peroxidation, protein synthesis inhibition and altered calcium load [4, 5]. Different studies have also shown anthracyclines causing altered gene expression [6]. We also noted that the LV GLS change occurred from the third month onward. In contrast, the ejection fraction changed only in the 6th month. By assessing regional myocardial function, strain serves as a potentially more sensitive indicator of early myocardial dysfunction [48]. Besides, strain is easy to use and reproducible. The various limitations in using LVEF for detecting myocardial damage include measurement errors, relatively late decline when considerable myocardial damage has already occurred, and dependence on preload and afterload. All these factors result in errors that affect the validity of LVEF in assessing early myocardial damage [47]. Besides, anthracyclines initially damage the endocardial layer. As endocardial layer is mainly formed by longitudinal fibers, the early endocardial dysfunction is best picked up by GLS rather than LVEF [49].

### Limitation

It is as single-center study with short duration of follow-up. There is uncertainty of long-term impact of early diastolic dysfunction. Besides, we did not use other imaging modalities (3D echo, cardiac MRI) and biomarkers (brain natriuretic peptide and troponin).

## Conclusions

Cardiotoxic chemotherapy is associated with significant diastolic and systolic left ventricular dysfunction. Ten percent of our cohort showed evidence of reduced LVEF at 6 months and one-third of patients showed diastolic dysfunction at 6 months. Increasing age and BMI were associated with early diastolic dysfunction. Diastolic dysfunction occurs early and predicts subsequent LV systolic dysfunction. Long-term studies are needed to determine whether early diastolic dysfunction is associated with long-term risk of heart failure.

## Data Availability

All data generated or analyzed during this study are included in this published article.

## Abbreviations

LVEF: Left ventricular ejection fraction
GLS: Global longitudinal strain
*E*: Inflow early diastolic velocity
*A*: Inflow late diastolic velocity
*e*′: Tissue Doppler mitral annular velocity
DT: Mitral valve deceleration time
IVRT: Isovolumic relaxation time
TR: Tricuspid regurgitation
